# Effects of *Clostridium butyricum* on Physiological Parameters and Gut Microbiota in Newborn Hanwoo Calves

**DOI:** 10.3390/ani15192785

**Published:** 2025-09-24

**Authors:** Min Ji Kim, Young Lae Kim, So Hee Lee, Jong Suh Shin, Sang Kook Kim, Soo An Kim, In Gi Jo, Gyung Hyun Jo, Seong Jeong Han, Ki Deuk Bae, Eu Jin Ban, Byung Ki Park

**Affiliations:** 1 Department of Animal Science, Kangwon National University, Chuncheon 24341, Republic of Korea; mjkim@kangwon.ac.kr (M.J.K.); dudfo123@naver.com (Y.L.K.); seesohev@naver.com (S.H.L.); jsshin@kangwon.ac.kr (J.S.S.); 2 Institute of R&D Center, Natural Good Things Inc., Seoul 28170, Republic of Korea; sehatkim@vitaminhouse.net (S.K.K.); sandy@naturalgoodthings.com (S.A.K.); jaimscho@hanmail.net (I.G.J.); jolldo@hanmail.net (G.H.J.); 3 Institute of R&D Center, Natural Pure Korea Inc., Damyang 57309, Republic of Korea; sjhan@npkor.co.kr (S.J.H.); busankd88@vitaminhouse.net (K.D.B.); jjbsj0030@npkor.co.kr (E.J.B.)

**Keywords:** *Clostridium butyricum*, gut microbiota, blood metabolism, Hanwoo calves

## Abstract

A healthy gut microbiota in calves is necessary for optimal performance and development. Considering that probiotics have been shown to improve gut microbiota, the aim of this study was to investigate the effects of *Clostridium butyricum* on growth performance, blood parameters, and gut microbiota in Hanwoo calves. In total, 92 calves from two farms were randomly assigned to four groups: a control and three treatment groups that received increasing doses of *C. butyricum* (CB1, CB2, and CB3) during the first 5 days after birth. Independent experiments were conducted at each farm with different measurements, where body weight was monitored and blood, rumen, and fecal samples were collected to assess physiological responses and microbial profiles. Notably, significant differences were observed in blood amylase and acid–base parameters, suggesting that *C. butyricum* supplementation may enhance metabolic stability and buffering capacity. Microbial profiling revealed preserved alpha diversity and compositional shifts in both the rumen and fecal microbiota. Particularly, there was an increase in the relative abundances of *Prevotella* and *Muribaculaceae* and a decrease in the abundances of the pathogenic genera *Escherichia* and *Shigella* in calves fed *C. butyricum*-supplemented diets. These changes, along with a trend toward a reduced frequency and severity of diarrhea, suggest that *C. butyricum* supplementation may support gut health and promote stable early growth in neonatal calves.

## Simple Summary

*Clostridium butyricum* possesses probiotic and metabolic properties. This study investigated the effects of *C. butyricum* on growth performance, gut microbiota, and various physiological parameters in Hanwoo calves. *C. butyricum* supplementation caused changes in blood amylase and acid–base parameters, suggesting improved metabolic stability and buffering capacity. Microbial analysis showed normal microbial diversity, increased abundance of beneficial microbes, and reduced levels of potential pathogens. Conclusively, *C. butyricum* may help establish a favorable intestinal environment in neonatal calves, supporting early gut health and disease prevention.

## 1. Introduction

Optimal feeding management is essential to reduce neonatal calf mortality and ensure healthy growth by minimizing pathogen exposure, promoting rumen development, and supporting the proliferation of beneficial microbiota [1,2,3]. Traditionally, antibiotics have been widely administered to prevent and treat diseases such as diarrhea in calves [4,5,6], and their use has also contributed to improved feed efficiency and growth performance [7,8,9]. Recently, the use of antimicrobial feed additives has been restricted/prohibited in several countries owing to concerns regarding antibiotic residues and environmental pollution [10,11]. Consequently, the identification of suitable antibiotic alternatives has attracted increasing interest. Probiotics, defined as live microorganisms such as lactic acid bacteria, yeasts, and butyrate-producing bacteria, have been investigated as promising alternatives to conventional antibiotics. Research findings indicate that probiotic supplementation in animal diets can support gut health and potentially enhance disease resistance [12,13,14]. 

Among the probiotic species, *Clostridium butyricum* has been recognized as a promising candidate. *C. butyricum* is a gram-positive, endophytic, anaerobic bacterium that produces short-chain fatty acids (SCFAs), particularly butyric acid. Unlike lactic acid bacteria, which require protective coatings to survive gastric conditions, *C. butyricum* naturally forms spores—highly resistant structures that enable the bacterium to withstand gastric acid, bile salts, digestive enzymes, and antibiotics, thereby maintaining its viability throughout the gastrointestinal tract [15,16]. As an anaerobe, *C. butyricum* primarily acts in the distal gut where oxygen levels are low, facilitating the growth of beneficial microbiota and suppressing harmful pathogens. Its fermentation metabolites, including SCFAs such as acetate, propionate, and butyrate, help maintain an acidic intestinal environment, which inhibits the colonization of pathogenic bacteria, supports the growth of beneficial microbes, and stimulates intestinal peristalsis, thereby promoting regular bowel movement [13,15,17]. Moreover, some studies have reported that butyrate downregulates the expression of various virulence-associated genes [18].

Several studies have investigated the effects of *C. butyricum* supplementation under practical conditions of livestock. Previous studies on the growth and gut health benefits of probiotics have mainly focused on monogastric animals [19,20,21,22,23,24]. In ruminants, *C. butyricum* supplementation has been reported to promote growth [25,26,27] and modulate the structure and composition of the gut microbiota [26,27]. Recent research has shown that *C. butyricum* supplementation during heat stress enhances rumen fermentation and alleviates the adverse physiological impacts of thermal stress [25]. Therefore, *C. butyricum* has the potential to improve the ruminal microbial environment, thereby contributing to improved productivity. However, most studies have been conducted in monogastric animals and adult ruminants, and there is limited information on the effects of *C. butyricum* supplementation in Hanwoo calves. Considering that calves have a relatively underdeveloped gastrointestinal microbiome, we hypothesized that the early introduction of beneficial microorganisms can improve their growth and overall health.

Therefore, the aim of this study was to investigate the effects of *C. butyricum* supplementation on growth performance, blood parameters, fecal microbiota composition, and disease incidence in Hanwoo calves, with a focus on promoting healthy early growth. Specifically, we designed experiments I and II to evaluate the effects of *C. butyricum* supplementation on growth performance and various health-related parameters in Hanwoo calves.

## 2. Materials and Methods

The current study was conducted from February to June 2024. All experimental protocols were approved by the Institutional Animal Care and Use Committee of Kangwon National University (Chuncheon, Republic of Korea) (protocol number: KW-250529-2), following the recommendations of the guidelines for animal research. Newborn Hanwoo calves (*n* = 92) were used, and experiments I (*n* = 52) and II (*n* = 40) were independently conducted on separate farms (hereafter referred to as Farm A and Farm B, respectively). Experiment I focused on body weight and rumen microbiota, whereas experiment II focused on blood parameters, fecal microbiota, and diarrhea frequency. Both experiments were designed to ensure an adequate sample size and uniform age distribution to minimize confounding factors unrelated to *C. butyricum* supplementation. Additionally, conducting multiple assessment protocols simultaneously can be stressful for newborn calves. Despite being conducted at different locations, both farms maintained the same feeding management practices and conditions to ensure consistency throughout the experiment.

### 2.1. Experiment I

#### 2.1.1. Experimental Design and Diets

Experiment I was performed to evaluate the effects of *C. butyricum* supplementation on body weight and ruminal microbiota in Hanwoo calves. The feeding trial was performed from March to August 2024 at Farm A using 52 newborn male calves. Newborn Hanwoo calves with an average body weight of 29.5 ± 4.2 kg (mean ± standard deviation) were randomly assigned to four treatment groups at birth (13 calves per group), with no significant difference in initial body weight among groups (*p* = 0.259): control (no supplementation; CON) and three *C. butyricum* supplementation groups receiving 10^8^ (CB1), 10^9^ (CB2), and 10^10^ (CB3) colony-forming units (CFU). For each supplemented group, a 2 g solid tablet containing the assigned concentration of *C. butyricum* with a corn starch-based excipient was dissolved in approximately 10 mL of water and administered orally for five consecutive days immediately after birth. Notably, the supplementation period was designed to target the early life stage when calves experience high microbial colonization to adapt to the external environment [28,29,30]. Our aim was to evaluate whether supplementation during this stage influences subsequent rumen fermentation and growth performance. Calves in the control group received an identical 2 g tablet containing only the excipient, administered at the same time and frequency as the treatment groups. All calves were housed with their dams in a communal rearing pen and were weaned at 3 months of age.

All the calves were fed 1.31 kg/day of calf starter in accordance with the Korean Feeding Standard for Hanwoo Steers [31]. They received food twice daily (at 08:00 and 17:00 h) and had ad libitum access to water. The ingredients and nutritional composition of the diets are listed in Table 1. The chemical composition of the experimental diets was analyzed using the methods recommended by the Association of Official Analytical Chemists [32]. Crude protein was determined using the Kjeldahl method, and crude fat by ether extraction. Crude fiber was analyzed using the Weende method, and ash content was determined by combustion at 550 °C. Moisture content was measured by oven drying at 105 °C. Neutral detergent fiber and acid detergent fiber were measured using the procedure described by Van Soest et al. [33].

#### 2.1.2. Body Weight and Average Daily Gain

Body weight was measured at birth and at 3 months of age. Additionally, the average daily gain (ADG) was calculated by subtracting birth weight from body weight at three months of age and dividing it by the total number of days in the experimental period.

#### 2.1.3. Rumen Fluid Sampling

Rumen fluid was collected at weaning (3 months of age) using an appropriate catheter, with three calves randomly selected from each group, to evaluate the effects of *C. butyricum* supplementation on rumen microbiota composition. Approximately 100 mL of rumen fluid was aseptically collected in a sterilized and immediately filtered through four layers of cheesecloth to remove the feed particles. Thereafter, the filtrate was stored at −80 °C until further analysis.

#### 2.1.4. 16S rRNA Gene Sequencing

Genomic DNA was extracted from the bacterial samples using the QIAamp Fast DNA Stool Mini Kit (Qiagen, Hilden, Germany) following the manufacturer’s protocol. Thereafter, the quantity and quality of the extracted DNA were assessed using a Nanodrop ND-1000 Spectrophotometer (Thermo Fisher Scientific, Wilmington, DE, USA). For 16S rRNA gene sequencing, the V3–V4 region of the bacterial 16S rRNA gene was amplified using MiSeq sequencing technology (Illumina, San Diego, CA, USA). A sequencing library was prepared according to the Illumina 16S Metagenomic Sequencing Library Preparation Protocol. All polymerase chain reactions (PCRs) for library preparation were conducted using 2X KAPA HiFi HotStart Ready Mix (Roche, Mannheim, Germany). The first PCR (1st PCR) was performed using universal primers with the following Illumina adapter overhang sequences: forward primer: 5′-TCGTCGGCAGCGTCAGATGTGTATAAGAGACAGCCTACGGGNGGCWGCAG-3′; reverse primer: 5′-GTCTCGTGGGCTCGGAGATGTGTATAAGAGACAGGACTACHVGGGTATCTAATCC-3′. The PCR conditions were as follows: initial denaturation at 95 °C for 30 s, followed by 25 cycles of denaturation at 95 °C for 30 s, annealing at 55 °C for 30 s, and extension at 72 °C for 30 s, with a final extension at 72 °C for 5 min. Amplified PCR products were purified using AMPure beads (Beckman Coulter, Brea, CA, USA). A second PCR (2nd PCR) was conducted to incorporate the Illumina index sequences using Nextera XT Indexed Primers (Illumina). The cycling conditions were identical to those of the first PCR, except for the number of cycles, which was reduced to 10. The final PCR products were purified using the AMPure beads. Purified libraries were quantified via qPCR according to the qPCR Quantification Protocol Guide (KAPA Library Quantification Kit for Illumina sequencing; Roche, Mannheim, Germany). Size distribution of the final libraries was assessed using a TapeStation D1000 ScreenTape (Agilent Technologies, Waldbronn, Germany). Sequencing was performed on the Illumina MiSeq platform (Illumina, San Diego, CA, USA) with a 2 × 300 bp paired-end sequencing strategy.

#### 2.1.5. Experiment I: Microbial Diversity and Functional Profiling

Briefly, the 16S amplicon sequence reads were analyzed using QIIME2 version 2024.10.1 (http://qiime.org/ accessed on 1 October 2024). The adapter and primer sequences were trimmed using the CutAdapt software version 4.9, and the chimeric sequences were filtered out. Denoising and merging were conducted using the plugin DADA2 to create an amplicon sequence variant (ASV) feature table. Taxonomic classifiers were manually constructed using the naïve Bayes classifier with the SILVA version 138 database. Thereafter, taxonomic assignment of ASVs was performed using the plugin q2-feature-classifier with 99% bacterial identity and representative sequences. Following the first taxonomic classification, an extra filtration step was used to obtain bacterial sequence data. Unassigned ASVs, chloroplasts, mitochondria, and non-bacterial taxa were excluded from the taxonomic filtration.

Additionally, alpha and beta diversities of the rumen and fecal microbiota were analyzed using QIIME 2 based on the ASV biological observation matrix (BIOM). Alpha diversity metrics included species richness (Chao1, ACE, observed ASVs, observed genera, and observed species) as well as Fisher’s, Shannon’s, and Simpson’s indices. Beta diversity was visualized using principal coordinate analysis (PCoA) based on the Bray–Curtis dissimilarity matrix. Hierarchical clustering was visualized using a dendrogram. All visualizations were generated using QIIME 2 artifacts (.qzv) and viewed using the QIIME 2 View web application (https://view.qiime2.org/, accessed on 10 September 2025). Diversity analyses were also conducted using MicrobiomeAnalyst based on the BIOM file. The same alpha diversity metrics were calculated, and PCoA and dendrograms were used to compare the microbial compositions. A Venn diagram was created using Venny software version 2.1 (https://bioinfogp.cnb.csic.es/tools/venny/, accessed on 17 July 2024) to compare shared and exclusive ASVs among the groups. Functional genetic profiling was performed using PICRUSt2 version 2.5.176. Moreover, the relative abundance of the MetaCyc pathway was used to infer functional differences among the treatment groups.

### 2.2. Experiment II

#### 2.2.1. Experimental Design and Diets

Experiment II was conducted to investigate the effects of *C. butyricum* supplementation on blood parameters, fecal scores, diarrhea frequency, and microbial diversity and functional profiles in Hanwoo calves. Feeding trial was performed from March to August 2024 at Farm B using 40 newborn male calves. Newborn Hanwoo calves were randomly selected and assigned to the experimental treatment groups at birth. All calves were then randomly allocated to four treatment groups (10 calves per group): a control group (no supplementation; CON), and three *C. butyricum* supplementation groups receiving 10^8^ (CB1), 10^9^ (CB2), and 10^10^ CFU (CB3). For each supplement group, a 2 g solid tablet containing the assigned concentration of *C. butyricum* was dissolved in approximately 10 mL of water and administered orally for five consecutive days immediately following birth. To prevent cross-contamination among treatments, the calves were kept in pens (three to four animals per pen) equipped with individual feed bins. All calves were nursed in the same pen as their dams and weaned at 3 months of age. Feeding management, diet formulation, and chemical analyses were performed as described in Experiment I. The nutritional composition of the calf starter is presented in Table 1.

#### 2.2.2. Blood Collection and Laboratory Analysis

Blood samples were collected from the jugular vein of 40 newborn calves at 7 d of age and immediately divided into heparin tubes containing EDTA (Becton Dickinson Vacutainer Systems, Franklin Lakes, NJ, USA) and serum tubes (Greiner BioOne, Kremsmünster, Austria). Thereafter, the collected blood samples were transported on ice to the laboratory, where hematological analysis and serum separation were performed via centrifugation (2000× *g*, 15 min, 4 °C).

Hematological parameters were measured using an automated hemocytometer (IDEXX Laboratories, Westbrook, ME, USA). The erythrogram included red blood cell count, hemoglobin concentration, hematocrit, mean cell volume (MCV), mean corpuscular hemoglobin concentration (MCHC), and reticulocyte count. Leukograms were assessed by determining the total white blood cell (WBC), neutrophil, lymphocyte, monocyte, eosinophil, and basophil counts, along with platelet counts. Blood biochemical parameters, including glucose, total cholesterol, triglycerides, amylase, non-esterified fatty acids (NEFAs), albumin, globulin, albumin/globulin ratio (A/G), total protein, total bilirubin, blood urea nitrogen (BUN), creatinine, gamma-glutamyl transferase (GGT), aspartate aminotransferase (AST), alkaline phosphatase (ALP), calcium, phosphorus, and magnesium, were analyzed using an automated dry biochemical analyzer (Boule Medical AB, Spanga, Sweden).

Blood gas parameters, including pH, partial pressure of oxygen (pO_2_), partial pressure of carbon dioxide (pCO_2_), bicarbonate (HCO_3_^−^), total carbon dioxide (TCO_2_), base excess (BE), and oxygen saturation (sO_2_), were analyzed using an automated blood gas analyzer (model, manufacturer, country) according to the manufacturer’s protocol. For quality control, calibration and internal standardization were performed before each set of measurements using certified reference materials provided by the manufacturer. All analyses were completed within 30 min of plasma separation to prevent gas diffusion and ensure data accuracy.

#### 2.2.3. Fecal Score and Diarrhea Frequency

Fecal scores and diarrhea frequency were recorded for each calf by trained evaluators to monitor the intestinal health and assess the effects of *C. butyricum* supplementation. Fecal scores were evaluated weekly before afternoon feeding using the four-point fecal scoring system described by Larson et al. [34]. The scoring system classified the fecal scores as follows: 1 = firm and well-formed, 2 = soft but maintained shape, 3 = loose and unformed, and 4 = watery diarrhea. Diarrhea was defined as a fecal score of 3 or higher, and the number of diarrhea days per calf was recorded throughout the experimental period. The total duration of diarrhea was calculated as the cumulative number of days for which each calf exhibited a fecal score of three or higher.

#### 2.2.4. Fecal Sampling, Microbial Diversity, and Functional Profiling

Fecal samples were collected from calves at 7 d of age, with three calves randomly selected from each group, to assess the effect of *C. butyricum* supplementation on gut microbiota. Approximately 10 g of feces were aseptically obtained from the rectum using sterile gloves, placed in sterile bags, transported on ice, and stored at −80 °C until analysis. Genomic DNA was extracted using a QIAamp PowerFecal Pro DNA Kit (Qiagen, Hilden, Germany). DNA amplification, sequencing, and downstream analyses were conducted using protocols identical to those described for rumen fluid in Experiment I, except for the sample type.

### 2.3. Statistical Analysis

Statistical analyses were performed using the R software (version 4.3.1; R Core Team, Vienna, Austria). Considering that the two experiments were conducted independently in different herds, each addressing distinct outcome variables, the datasets were analyzed separately. For each body weight and blood metabolite parameter, data were assessed for normality using the Shapiro–Wilk test and for homogeneity of variance using Levene’s test. When both the normality and homogeneity of variance assumptions were satisfied, a Type II analysis of variance (ANOVA) was conducted, and multiple comparisons were performed using Tukey’s honest significant difference (HSD) test. If the normality assumption was met but the variances were heterogeneous, Welch’s ANOVA was used. For non-normally distributed data, the Kruskal–Wallis test was applied, followed by Dunn’s test for multiple comparisons, with Bonferroni correction. In addition, estimated marginal means were used to perform planned contrasts between the control group and the average of the groups supplemented with *C. butyricum*. Subsequently, polynomial contrasts were applied to test for linear and quadratic effects of supplementation. For nonparametric data, aligned rank transform models were applied to enable factorial contrasts, and robust standard errors were used when appropriate. Descriptive statistics were expressed as group means, standard errors of the mean (SEMs), and sample sizes. Statistical significance was set at *p* < 0.05, and tendencies were considered at 0.05 ≤ *p* ≤ 0.10.

## 3. Results and Discussions

### 3.1. Body Weight and Average Daily Gain

Table 2 shows the effects of *C. butyricum* on the body weight, ADG, and FCR of Hanwoo calves. Birth and weaning weights were not significantly affected by *C. butyricum* supplementation (birth weight, *p* = 0.259; weaning weight, *p* = 0.896). Although ADG showed an increasing trend with increasing levels of *C. butyricum* supplementation, ADG was not significantly different (*p* = 0.835) among the groups, with values ranging from 0.86 to 0.91 kg/day.

Research findings indicate that the effects of *C. butyricum* supplementation on growth performance vary among different livestock species and management conditions. For example, Li et al. [26] found that *C. butyricum* supplementation significantly increased DMI and body weight in cows, whereas Zhang et al. [27] found no significant changes in the growth indicators in goats. These differences suggest that the effects of *C. butyricum* supplementation may be affected by various factors, including species, current health status, feeding environment, and composition of gut microbes.

In the present study, *C. butyricum* supplementation did not considerably affect growth performance parameters, including birth weight, weaning weight, and ADG, in Hanwoo calves. This may reflect the limited direct impact of probiotics on growth, although it is also possible that factors such as dosage, trial duration, and sample size may have influenced the statistical outcomes. Therefore, future studies should adopt longer feeding trials, include a range of dosage levels, and ensure adequate sample sizes to determine the effect of *C. butyricum* on the growth performance of calves.

### 3.2. Blood Biochemical Parameters

Table 3 shows the effects of *C. butyricum* supplementation on blood metabolite levels. Blood amylase was markedly lower in the CB1 and CB2 groups compared with the CON group (overall, *p* < 0.01), whereas the CB3 group showed intermediate values. Triglyceride concentrations also exhibited a quadratic response (*p* = 0.036), being higher in the CB1 and CB2 groups but declining in the CB3 group. Other blood metabolites, including cholesterol, NEFA, total protein, albumin, and liver enzymes (AST, GGT, ALP), did not differ significantly among groups (*p* > 0.05).

Amylase is a digestive enzyme primarily secreted by the pancreas and salivary glands and is present at high concentrations in digestive fluids, with small amounts detected in the blood and urine [35,36,37]. In ruminant species, salivary secretion of amylase is negligible, distinguishing them from non-ruminant animals [38,39,40]. Circulating amylase levels are commonly utilized as biomarkers for pancreatic and gastrointestinal functions and have been employed in the clinical evaluation of pathological conditions such as pancreatitis and gastroenteritis.

Recently, there has been growing recognition that indicators such as serum amylase should be interpreted within the framework of host–microbiota interactions, particularly along the gut–pancreas axis. This concept has led to increasing research interest on the bidirectional physiological mechanisms underlying this axis. Among various microbial metabolites, VFAs have been reported to influence pancreatic function [12,41]. Particularly, sodium butyrate modulates pancreatic function by suppressing nuclear factor-kappa B (NF-κB) activity and inhibiting histone deacetylase, thereby regulating inflammation and fibrosis [41,42].

In the present study, the observed decrease in serum amylase concentration following *C. butyricum* supplementation suggests that changes in the intestinal environment indirectly influence pancreatic function via the gut–pancreas axis. However, ruminants possess a complex microbial ecosystem within the rumen and distinct microbial communities along the intestinal tract, resulting in a multilayered microbiota structure. Because of this complexity, the physiological link between microbial populations and digestive organs, including the pancreas, is highly intricate and not well understood in ruminants. Furthermore, as this study did not directly assess pancreatic responses or the expression of genes related to pancreatic function, definitive conclusions regarding the underlying mechanisms of the observed phenomenon cannot be drawn.

However, previous studies have reported that metabolites, such as VFAs, can improve intestinal homeostasis, modulate immune and metabolic functions, and potentially affect extraintestinal organs such as the pancreas [23,24,25]. Our findings may provide foundational evidence for future investigations into microbiota–organ interactions mediated by the gut–pancreas axis in ruminants. However, further studies are required to elucidate the precise biological mechanisms underlying this process.

Table 4 shows the hematological parameters of calves fed diets supplemented with different *C. butyricum* doses. No significant differences were observed among the groups for most parameters. In contrast, the white blood cell count tended to differ (overall, *p* = 0.078; linear, *p* = 0.072) and showed a significant quadratic trend (*p* = 0.014), increasing in the CON group, decreasing in the CB1 and CB2 groups, and then increasing in the CB3 group. Neutrophil counts were significantly higher in the CON group than in the CB1 and CB2 groups (overall, *p* = 0.033; quadratic response [*p* = 0.006]), with the CB3 group showing intermediate values.

Notably, differences in WBC and neutrophil counts were observed among the treatment groups. Although the mechanisms of *C. butyricum* in immune regulation and inflammation were not directly investigated in the present study, previous studies have reported that *C. butyricum* exhibits anti-inflammatory and immunomodulatory effects by regulating cytokine production and immune cell differentiation [43,44]. Specifically, *C. butyricum* enhances the host immune system by upregulating pro-inflammatory cytokines such as IL-8, IL-6, and TNF-α, and also exerts beneficial effects through the production of anti-inflammatory cytokines such as IL-10 [45,46]. Additionally, some studies have shown that *C. butyricum* may suppress the NF-κB signaling pathway, thereby regulating the transcription of inflammatory genes and inhibiting excessive inflammatory responses and immune cell activation [42,47]. Similarly, Zhang et al. [21] reported that dietary supplementation with *C. butyricum* upregulated TNF-α and IL-4 concentrations in the jejunal mucosa of broiler chickens compared with those in the control group. Additionally, studies in piglets have shown that 0.4% *C. butyricum* supplementation increases the relative mRNA expression of TLR2 and IL-10 in the ileum. Although these findings were obtained from monogastric animals, they suggest that *C. butyricum* supplementation may modulate immune responses by balancing pro-inflammatory and anti-inflammatory signaling pathways, contributing to improved intestinal immune regulation. However, a limitation of this study is that specific immune-related markers, such as cytokine levels, were not analyzed. Future studies should incorporate comprehensive assessments of inflammation-related markers and hematological parameters to elucidate the immunomodulatory effects of *C. butyricum*.

Furthermore, the effects of *C. butyricum* supplementation on blood acid–base parameters are presented in Table 5. No significant differences were observed in pH levels among the groups (overall, *p* > 0.05). However, there was a significant increase in TCO_2_, HCO_3_, and base excess and a decrease in anion gap in the CB3 group compared with those in the CON group (overall, *p <* 0.01). Additionally, these parameters showed clear linear responses across the supplementation levels (linear, *p* < 0.01).

TCO_2_, HCO_3_^−^, and pH are critical indicators of systemic acid–base balance. Among these, HCO_3_^−^—which constitutes the major component of TCO_2_—functions as a primary physiological buffer and is widely recognized as a key marker for assessing metabolic acid–base disorders, including metabolic acidosis and alkalosis. The bicarbonate buffering system not only maintains systemic acid–base homeostasis but also plays a vital role in regulating the ruminal environment of gastrointestinal regions with active microbial fermentation [48,49,50]. In the rumen, the accumulation of VFAs during fermentation predisposes the environment to acidification; however, HCO_3_^−^ secreted via saliva contributes to the neutralization of this acidity, thereby stabilizing ruminal pH. Consequently, HCO_3_^−^ and VFAs in the rumen are essential for sustaining microbial homeostasis and acid–base equilibrium. Moreover, localized buffering activity in the rumen may have systemic implications, potentially influencing circulating HCO_3_^−^ concentrations [51,52,53].

In the present study, *C. butyricum* supplementation significantly increased both serum HCO_3_^−^ concentration and blood pH. Although *C. butyricum* was not directly investigated in previous ruminal acidosis models, the existing literature has demonstrated that co-administration of probiotics and sodium bicarbonate ameliorates acid–base disturbances and elevates blood pH under experimentally induced acidosis [14]. Collectively, these findings imply that probiotics may contribute to mitigating acidic ruminal conditions and restoration of systemic pH by modulating SCFA production and enhancing ruminal buffering capacity.

Therefore, these findings suggest that *C. butyricum* supplementation may influence the profile of ruminal fermentation metabolites, thereby enhancing the activity of the bicarbonate buffering system and promoting ruminal pH stability, which may contribute to the maintenance of systemic acid–base homeostasis.

### 3.3. Diarrhea Frequency and Severity

Table 6 shows the effects of *C. butyricum* supplementation on the frequency and severity of diarrhea in Hanwoo calves. Although overall group differences were not significant (*p >* 0.05), both parameters showed a tendency to decrease linearly with increasing supplementation levels (frequency, *p* = 0.051; severity, *p* = 0.052). The highest frequency and severity were observed in the CON group (5.90 and 2.00, respectively), whereas the lowest values were recorded in the CB3 group (3.10 and 1.10, respectively).

Although the reductions in diarrhea frequency and severity were not statistically significant, the numerical trends suggest that *C. butyricum* supplementation may play a beneficial role in mitigating gastrointestinal disturbances in young calves. These observations are consistent with previous findings showing that butyrate-producing probiotics can enhance gut barrier integrity, modulate local immune responses, and suppress pathogenic bacteria, thereby reducing diarrhea incidence and severity [19,54]. Additionally, the observed dose-dependent decline, particularly in the CB3 group, supports the hypothesis that higher levels of *C. butyricum* may effectively stabilize the gastrointestinal environment during the early growth phase. Moreover, the lack of statistical significance may be attributed to sample size limitations or variability in individual responses; however, these findings warrant further investigation under controlled conditions in larger cohorts.

### 3.4. Rumen and Fecal Alpha Diversity and Microbiota 

In this study, we investigated the alpha diversities of the rumen and fecal microbiota of calves fed *C. butyricum*-supplemented diets (Table 7). No significant differences were observed among the treatment groups for any of the indices evaluated, including the ASVs, Chao1, Shannon, and Gini–Simpson indices, in both the rumen and feces (*p* > 0.05). In the rumen, the Shannon index ranged from 5.84 to 6.70, and the Gini–Simpson index remained below 0.05 across all groups. In fecal samples, the Shannon index was consistently above 5, and the Gini–Simpson index ranged from 0.059 to 0.121, suggesting a more even microbial community than that in the rumen. Overall, these values indicate that the ecological stability of microbial diversity and evenness was preserved despite probiotic supplementation.

**Figure 1:**
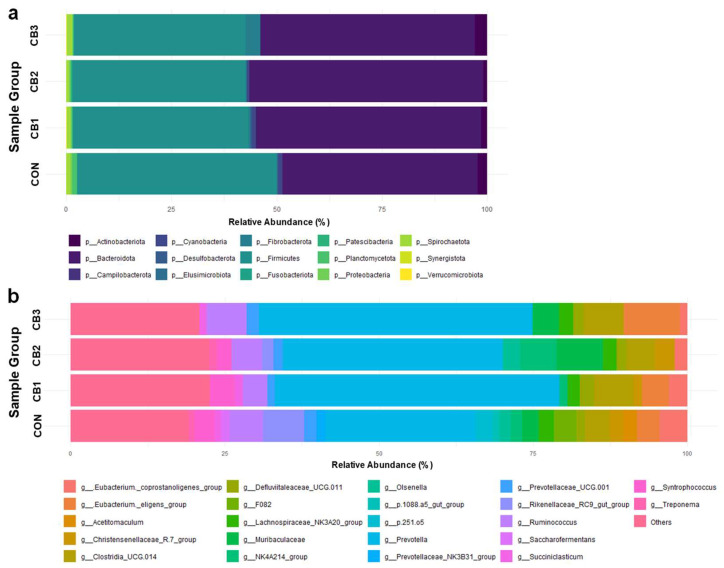
Effects of *C. butyricum* on the rumen microbiota composition in Hanwoo calves. CON = control group (no supplementation, *n* = 3); CB1 = *C. butyricum* 10^8^ CFU (*n* = 3); CB2 = *C. butyricum* 10^9^ CFU (*n* = 3); CB3 = *C. butyricum* 10^10^ CFU (*n* = 3). The relative abundances of (**a**) group average of phyla and (**b**) the group average of genera are visualized. Genera with a relative abundance in the bottom 20% and phyla with a relative abundance below 1% were grouped under “Others”.

Additionally, the composition of rumen microbiota is summarized in Figure 1 and Tables S1 and S2. At the phylum level, Firmicutes and Bacteroidetes were predominant across all groups, accounting for approximately 40–51% and 41–55% of the total sequences, respectively. Other phyla, including Actinobacteria, Fibrobacterota, Cyanobacteria, and Spirochaetota, were detected at lower relative abundances (<5%), with no significant differences among the groups (*p* > 0.05). Additionally, similarities and differences in community structure among groups were determined and visualized using principal coordinate analysis (PCoA) based on beta diversity metrics (Supplementary Figure S1).

At the genus level, *Prevotella* was the most dominant taxon, with a relative abundance ranging from 24.13% in the CON group to 45.93% in the CB2 group. Although these differences were not statistically significant (*p* = 0.238), higher abundances were observed in all *C. butyricum*-supplemented groups than in the CON group. Similarly, *Muribaculaceae* abundance increased from 2.95% in the CON group to 7.52% in the CB3 group, showing a near-significant trend (*p* = 0.056). In contrast, the relative abundance of the *Rikenellaceae* RC9 gut and F082 decreased notably in the supplementation groups.

*Prevotella* plays a central role in carbohydrate and hydrogen metabolism in the rumen of ruminants. Members of this genus possess a wide range of enzymes that are capable of degrading various polysaccharides, thereby contributing to VFA production [55,56]. Additionally, some species have been reported to be proteolytic bacteria that produce cysteine proteases and collagen-degrading enzymes [55,57]. Although the functional importance of *Prevotella* has been well demonstrated in previous studies, the present study did not include metabolomic analyses, such as VFA profiling, to directly assess its metabolic activity or its contribution to fermentation. In future studies, metabolomic approaches should be integrated to quantitatively evaluate the effects of *C. butyricum* on rumen fermentation parameters and clarify its relationship with microbial composition.

In the present study, an increasing trend in the relative abundances of not only *Prevotella* but also *Muribaculaceae* was observed in the *C. butyricum*-supplemented groups. *Muribaculaceae*, primarily belonging to the phylum Bacteroidetes, is a group of commensal bacteria that produces enzymes specialized in degrading dietary polysaccharides and fibers [58,59]. Their abundance has been shown to vary in response to the dietary forage-to-concentrate ratio, specific feed ingredients, and probiotic supplementation [60,61] and they may interact with fermentation conditions and rumen microbial community structure in response to environmental changes [62,63].

Previous studies have reported that dietary supplementation with *C. butyricum* increases the relative abundance of genera such as *Prevotella*, *Ruminococcaceae*, and *Megasphaera* and promotes the production of VFAs [23,27]. Moreover, appropriate levels of *C. butyricum* supplementation can facilitate probiotic colonization of the gastrointestinal tract, enhance gut barrier function, and protect against pathogenic bacterial invasion [24]. In the present study, all groups were fed an identical total mixed ration and maintained under standardized housing conditions to minimize the effects of external factors. Under these controlled conditions, the observed increase in the relative abundances of *Prevotella* and *Muribaculaceae* in the *C. butyricum*-supplemented groups suggests that this strain may indirectly promote the growth of these microbes by improving the gut microenvironment, reducing the abundance of competing bacteria, and altering the availability of substrates.

**Figure 2:**
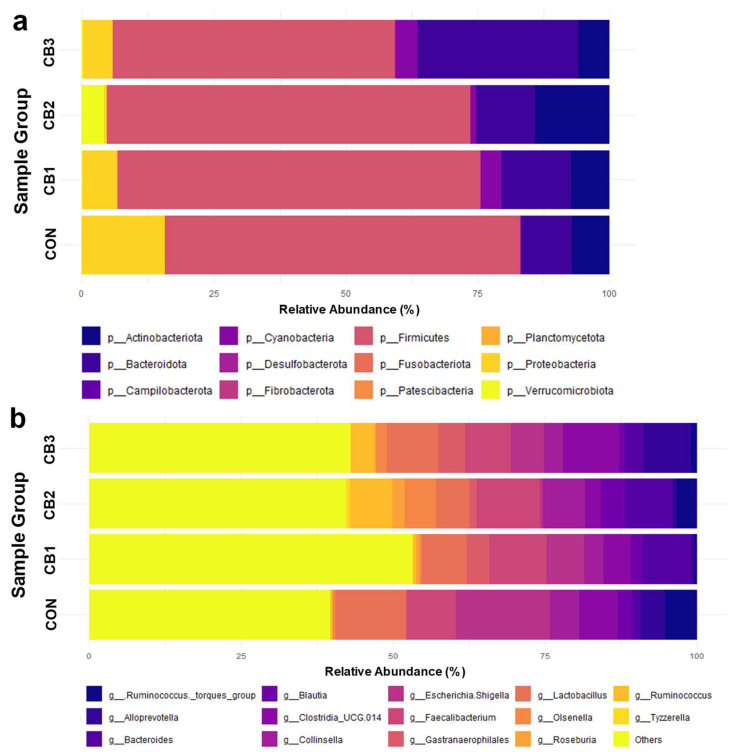
Effects of *C. butyricum* on fecal microbiota composition in Hanwoo calves. CON = control group (no supplementation, *n* = 3); CB1 = *C. butyricum* 10^8^ CFU (*n* = 3); CB2 = *C. butyricum* 10^9^ CFU (*n* = 3); CB3 = *C. butyricum* 10^10^ CFU (*n* = 3). The relative abundances of (**a**) the group average of phyla and (**b**) the group average of genera are visualized. Genera with a relative abundance in the bottom 20% and phyla with a relative abundance below 1% were grouped under “Others”.

Furthermore, the composition of fecal microbiota is summarized in Figure 2 and Tables S3 and S4. At the phylum level, Firmicutes was the most dominant taxon across all groups, accounting for approximately 63–71% of the total sequences, followed by Proteobacteria (4.8–18.2%), Bacteroidetes (10.6–31.9%), and Actinobacteria (5.6–11.8%). Other phyla, such as Cyanobacteria and Verrucomicrobiota, were present in lower proportions (<5%), with no significant differences among the treatment groups (*p* > 0.05). Moreover, similarities and differences in community structure among groups were determined and visualized using PCoA based on beta diversity metrics (Supplementary Figure S2).

Similarly, no significant differences (*p* > 0.05) were observed in the relative abundances of most genera among the treatment groups. However, several genera showed trends in response to *C. butyricum* supplementation. *Escherichia*–*Shigella* showed a decreasing trend, with the highest abundance observed in the control group (13.78%) and progressively lower values in the treatment groups, reaching 2.31% in CB3. In contrast, increasing trends were observed for genera such as *Bacteroides*, *Faecalibacterium*, and *Gastranaerophilales* in the *C. butyricum*-supplemented group. Although *Tyzzerella* showed a significant increase in CB3, its overall abundance was low, suggesting that its biological relevance is limited.

The reduced abundance of *E*. *coli* in the *C. butyricum*-supplemented groups suggests that the probiotic suppresses potentially pathogenic bacteria. The *Escherichia*–*Shigella* genera includes several strains known for their pathogenicity, such as enterotoxigenic *E*. *coli* (ETEC), which is a major cause of diarrhea and gastrointestinal disturbances in animals [64,65]. These pathogens can cause diarrhea, dehydration, and impaired nutrient absorption, ultimately leading to reduced growth and increased mortality in neonatal calves. Therefore, the observed decrease in *E*. *coli* abundance following *C. butyricum* supplementation may be associated with its benefits in reducing the frequency and severity of calf diarrhea.

## 4. Conclusions

In this study, we investigated the effects of *C. butyricum* supplementation on growth performance, blood parameters, and gut microbiota composition in Hanwoo calves. Although no significant differences were observed in growth performance, calves in the *C. butyricum*-supplemented groups exhibited a tendency toward increased weight gain compared with those in the control group. Physiological responses were also detected in certain blood parameters and acid–base indicators, particularly in the *C. butyricum*-supplemented groups, suggesting improved buffering capacity and enhanced metabolic stability. Microbial analysis showed normal alpha diversity and notable shifts in both rumen and fecal microbiota compositions in response to *C. butyricum* supplementation. Specifically, the relative abundances of beneficial genera, such as *Prevotella* and *Muribaculaceae*, tended to increase, whereas potentially pathogenic *Escherichia* and *Shigella* showed a decreasing trend. Overall, these findings suggest that *C. butyricum* supplementation may modulate the ruminal environment and microbial composition to support gut health and immune balance in calves. Additionally, the observed trend toward reduced diarrhea frequency and severity indicates the potential benefits of promoting intestinal stability and supporting early growth in neonatal calves. Among the treatment groups, the highest dosage (10^10^ CFU, CB3) appeared to induce the most pronounced physiological and microbial changes. However, this study did not include a dose–response analysis or follow-up trials to determine the optimal dosage, highlighting the need for further investigation. Moreover, this study was limited to the early growth phase and did not include direct profiling of rumen fermentation metabolites. Additionally, the number of sampling points was restricted, preventing repeated measurements over time. To fully understand the long-term benefits and microbial mechanisms of *C. butyricum* supplementation, future studies should include extended growth monitoring, repeated sampling designs, and integrative analyses such as metabolomic profiling and microbial co-occurrence network analysis.

## Figures and Tables

**Table 1:** Chemical composition of experimental diets for Hanwoo calves (DM basis).

Items	Contents	
	Concentrate	Timothy
Nutrient composition (%)		
Dry matter	91.40 ± 0.76	93.60 ± 0.81
Crude protein	19.23 ± 0.43	13.25 ± 0.32
Ester extract	3.12 ± 0.73	1.50 ± 0.12
Crude ash	8.59 ± 0.86	6.84 ± 0.11
Crude fiber	12.29 ± 4.11	43.06 ± 0.55
Neutral detergent fiber	34.54 ± 3.74	65.17 ± 0.93
Acid digestible fiber	17.89 ± 1.52	38.35 ± 0.43

Values are presented as mean ± standard deviation.

**Table 2:** Effects of *C. butyricum* on body weight in Hanwoo calves.

Item	CON	CB1	CB2	CB3	SEM	*p*-Value			
						Overall	Linear	Quadratic	CON vs. CB
Birth	30.23	31.00	27.92	29.00	0.57	0.259	0.195	0.894	0.491
Weaning	91.46	92.38	93.31	95.08	1.71	0.896	0.452	0.903	0.598
Average daily gain	0.86	0.88	0.90	0.91	0.02	0.835	0.183	0.964	0.26

CON: control group (no supplementation, *n* = 13); CB1: *C. butyricum* 10^8^ CFU (*n* = 13); CB2: *C. butyricum* 10^9^ CFU (*n* = 13); CB3: *C. butyricum* 10^10^ CFU (*n* = 13). Overall: main effect of treatment; Linear: linear trend across supplementation levels; Quadratic: quadratic trend; CON vs. CB: contrast between the control group and the mean of all *C. butyricum* groups.

**Table 3:** Effects of *C. butyricum* on blood metabolites in Hanwoo calves.

Item	CON	CB1	CB2	CB3	SEM	*p*-Value			
						Overall	Linear	Quadratic	CON vs. CB
Glucose (mmol/L)	7.56	6.98	6.94	7.37	0.14	0.370	0.628	0.092	0.169
Total cholesterol (mg/dL)	92.92	107.69	93.39	105.18	5.45	0.695	0.651	0.895	0.477
Triglycerides (mg/dL)	36.30	58.74	53.78	47.99	3.99	0.158	0.630	0.036	0.077
Amylase (U/L)	169.70 ^a^	111.40 ^b^	123.89 ^b^	133.10 ^a^	5.14	<0.010	<0.010	<0.010	<0.010
NEFA (mEq/L)	0.21	0.24	0.21	0.23	0.01	0.553	0.710	0.913	0.476
Albumin (g/dL)	3.37	3.37	3.31	3.32	0.03	0.842	0.437	0.941	0.601
Globulin (g/dL)	3.84	3.86	4.12	4.33	0.10	0.205	0.043	0.617	0.223
A/G	0.91	0.88	0.83	0.78	0.03	0.170	0.049	0.700	0.287
Total protein (g/dL)	7.22	7.27	7.44	7.63	0.09	0.359	0.085	0.707	0.273
Total bilirubin (mg/dL)	0.28	0.18	0.16	0.17	0.03	0.772	0.467	0.491	0.371
BUN (mg/dL)	13.68	9.09	9.64	9.95	0.74	0.347	0.545	0.190	0.136
Creatinine (µmol/L)	86.20	92.03	85.31	82.03	2.40	0.525	0.375	0.353	0.963
GGT (U/L)	47.90	47.30	62.00	68.40	4.84	0.352	0.086	0.708	0.249
AST (U/L)	60.20	44.50	40.22	43.50	3.15	0.532	0.276	0.393	0.227
ALP (U/L)	589.30	774.50	580.33	689.50	44.47	0.373	0.788	0.671	0.371
Calcium (mg/dL)	10.48	10.72	10.72	10.82	0.14	0.849	0.422	0.802	0.404
Phosphorus (mg/dL)	8.06	8.52	7.05	7.53	0.21	0.078	0.092	0.970	0.430
Magnesium (mg/dL)	1.65	1.76	1.55	1.66	0.08	0.331	0.152	0.673	0.551

CON: control group (no supplementation, *n* = 10); CB1: *C. butyricum* 10^8^ CFU (*n* = 10); CB2: *C. butyricum* 10^9^ CFU (*n* = 10); CB3: *C. butyricum* 10^10^ CFU (*n* = 10). Overall: main effect of treatment; Linear: linear trend across supplementation levels; Quadratic: quadratic trend; CON vs. CB: contrast between the control group and the mean of all *C. butyricum* groups. Different superscript letters within a row indicate significant differences (*p* < 0.05). NEFA: non-esterified fatty acids; A/G: albumin to globulin ratio; BUN: blood urea nitrogen; GGT: gamma-glutamyl transferase activity; AST: aspartate aminotransferase activity; ALP: alkaline phosphatase activity.

**Table 4:** Effects of *C. butyricum* on hematological parameters in Hanwoo calves.

Item	CON	CB1	CB2	CB3	SEM	*p*-Value			
						Overall	Linear	Quadratic	CON vs. CB
Red blood cell (M/µL)	9.27	8.95	7.90	8.20	0.22	0.094	0.027	0.466	0.062
Hematocrit (%)	32.43	30.97	27.31	30.23	0.85	0.208	0.174	0.199	0.135
Hemoglobin (g/dL)	10.55	9.92	9.00	9.60	0.23	0.130	0.067	0.181	0.051
MCV (fL)	34.90	34.64	34.37	36.89	0.38	0.073	0.085	0.063	0.634
MCHC (g/dL)	32.82	32.01	33.38	31.89	0.33	0.459	0.629	0.611	0.605
RDW (%)	39.85	39.74	38.41	37.62	0.44	0.201	0.041	0.692	0.206
Reticulocyte (K/µL)	1.81	0.61	1.63	2.05	0.28	0.094	0.322	0.025	0.452
White blood cell (K/µL)	15.83 ^a^	10.44 ^b^	10.71 ^b^	14.68 ^ab^	0.72	0.015	0.578	0.001	0.012
Neutrophil (K/µL)	9.87 ^a^	5.60 ^ab^	4.81 ^b^	7.84 ^ab^	0.67	0.033	0.223	0.005	0.016
Eosinophils (K/µL)	0.04	0.03	0.02	0.05	0.21	0.288	0.436	0.083	0.701
N/E	2.40	1.51	1.02	1.46	0.23	0.198	0.231	0.130	0.129
Lymphocyte (K/µL)	4.94	4.06	4.91	5.48	0.06	0.100	0.171	0.076	0.777
Monocytes (K/µL)	0.77	0.64	0.77	0.93	0.005	0.588	0.460	0.511	0.961
Basophils (K/µL)	0.21	0.11	0.19	0.25	0.06	0.912	0.925	0.846	0.720
Platelets (K/µL)	933.40	952.80	992.44	1027.00	33.98	0.778	0.307	0.914	0.478

CON: control group (no supplementation, *n* = 10); CB1: *C. butyricum* 10^8^ CFU (*n* = 10); CB2: *C. butyricum* 10^9^ CFU (*n* = 10); CB3: *C. butyricum* 10^10^ CFU (*n* = 10). Overall: main effect of treatment; Linear: linear trend across supplementation levels; Quadratic: quadratic trend; CON vs. CB: contrast between the control group and the mean of all *C. butyricum* groups. Different superscript letters within a row indicate significant differences (*p* < 0.05). M/µL: million per microliter; K/µL: thousand per microliter; MCV: mean corpuscular volume; MCHC: Mean corpuscular hemoglobin concentration; RDW: Red cell distribution width; N/E: neutrophil-to-eosinophil ratio.

**Table 5:** Effects of *C. butyricum* on blood acid base parameters in Hanwoo calves.

Item	CON	CB1	CB2	CB3	SEM	*p*-Value			
						Overall	Linear	Quadratic	CON vs. CB
TCO_2_ (mmol/L)	24.70 ^b^	32.90 ^ab^	33.78 ^ab^	35.90 ^a^	1.16	0.020	0.002	0.144	0.004
HCO_3_	23.01 ^b^	30.94 ^ab^	31.87 ^ab^	33.86 ^a^	1.14	0.025	0.002	0.148	0.005
pH	7.20	7.29	7.30	7.32	0.01	0.057	0.007	0.714	0.042
Base excess (mmol/L)	−5.10 ^b^	4.40 ^ab^	5.33 ^ab^	7.80 ^a^	1.37	0.032	0.003	0.155	0.007
Anion gap (mmol/L)	16.50 ^a^	14.70 ^ab^	13.11 ^b^	13.4 ^b^	0.32	<0.010	0.000	0.040	<0.010

CON: control group (no supplementation, *n* = 10); CB1: *C. butyricum* 10^8^ CFU (*n* = 10); CB2: *C. butyricum* 10^9^ CFU (*n* = 10); CB3: *C. butyricum* 10^10^ CFU (*n* = 10). Overall: main effect of treatment; Linear: linear trend across supplementation levels; Quadratic: quadratic trend; CON vs. CB: contrast between the control group and the mean of all *C. butyricum* groups. Different superscript letters within a row indicate significant differences (*p* < 0.05). TCO_2_: total carbon dioxide; HCO_3_: bicarbonate.

**Table 6:** Effects of C. butyricum on diarrhea frequency and severity in Hanwoo calves.

Item	CON	CB1	CB2	CB3	SEM	*p*-Value			
						Overall	Linear	Quadratic	CON vs. CB
Frequency	5.90	4.70	4.67	3.10	0.51	0.223	0.051	0.765	0.132
Severity	2.00	1.10	1.22	1.10	0.16	0.108	0.052	0.211	0.015

CON: control group (no supplementation, *n* = 10); CB1: *C. butyricum* 10^8^ CFU (*n* = 10); CB2: *C. butyricum* 10^9^ CFU (*n* = 10); CB3: *C. butyricum* 10^10^ CFU (*n* = 10). Overall: main effect of treatment; Linear: linear trend across supplementation levels; Quadratic: quadratic trend; CON vs. CB: contrast between the control group and the mean of all *C. butyricum* groups.

**Table 7:** Effects of *C. butyricum* on alpha diversity of microbiota in Hanwoo calves.

Items	CON	CB1	CB2	CB3	SEM	*p*-Value
Rumen						
ASVs	431.67	464.33	405.00	446.00	66.95	0.973
Chao1	464.91	505.04	441.89	484.65	56.08	0.972
Shannon	6.70	6.39	5.84	6.26	0.26	0.776
Gini–Simpson	0.023	0.046	0.047	0.036	0.010	0.703
Feces						
ASVs	145.75	153.5	159.75	232.25	15.57	0.254
Chao1	155.58	159.11	173.89	257.73	14.18	0.196
Shannon	6.70	6.39	5.84	6.26	0.30	0.509
Gini–Simpson	0.121	0.076	0.109	0.059	0.020	0.644

CON: control group (no supplementation); CB1: *C. butyricum* 10^8^ CFU; CB2: *C. butyricum* 10^9^ CFU; CB3: *C. butyricum* 10^10^ CFU. Rumen samples: *n* = 13 per group; Feces samples: *n* = 10 per group. ASVs: amplicon sequence variant; SEM: standard error of the mean.

## Data Availability

The data supporting this study are not publicly available due to restrictions imposed by the funding agency and contractual/ethical obligations with participating farms. Limited, de-identified data may be shared upon reasonable request for non-commercial academic purposes only, subject to prior approval by the corresponding author and the funding institution.

## Supplementary Materials

The following supporting information can be downloaded at: https://www.mdpi.com/article/10.3390/ani15192785/s1, Figure S1: Principal coordinate analysis (PCoA) of ruminal microbiota in Hanwoo calves supplemented with *C. butyricum*; Figure S2: Principal coordinate analysis (PCoA) of fecal microbiota in Hanwoo calves supplemented with *C. butyricum*; Table S1: Effects of *C. butyricum* on the rumen microbiota composition (phylum level) in Hanwoo calves; Table S2: Effects of *C. butyricum* on the rumen microbiota composition (genus level) in Hanwoo calves; Table S3: Effects of *C. butyricum* on the fecal microbiota composition (phylum level) in Hanwoo calves; Table S4: Effects of *C. butyricum* on the rumen microbiota composition (genus level) in Hanwoo calves.

## Abbreviations

The following abbreviations are used in this manuscript:

SCFA	Short chain fatty acid
ADG	Average daily gain
FCR	Feed conversion ratio
DMI	Dry matter intake
MCV	Mean cell volume
MCHC	Mean corpuscular hemoglobin concentration
WBC	White blood cell
NEFA	Non-esterified fatty acids
BUN	Blood urea nitrogen
GGT	Gamma-glutamyl transferase
AST	Aspartate aminotransferase
ALP	Alkaline phosphatase
