# Supplementary material for: Effects of Clostridium butyricum on Physiological Parameters and Gut Microbiota in Newborn Hanwoo Calves
Source: Animals (Basel). 2025 Sep 24;15(19):2785. doi: 10.3390/ani15192785 (PMC12524223; doi:10.3390/ani15192785)
Supplement: Supplementary file 1 [file animals-15-02785-s001.zip › animals-3849209-supplementary.pdf]

Suppementary Table S1. Effects of C. butyricum on the rumen microbiota composition (phylum level) in Hanwoo calves

Items (%)	CON	CB1	CB2	CB3	SEM	p-value
Actinobacteria	2.39	1.19	0.94	2.74	0.41	0.346
Bacteroidetes	41.56	52.55	55.49	50.40	3.48	0.589
Cyanobacteria	1.50	0.80	0.54	0.04	0.36	0.611
Firmicutes	51.43	42.20	41.29	39.88	2.96	0.566
Fibrobacterota	0.27	0.92	0.24	4.76	1.00	0.358
Spirochaetota	1.43	1.24	0.66	1.54	0.33	0.837
Others	1.43	1.11	0.84	0.64	0.21	0.620

CON = control group (no supplementation, n = 3); CB1 = C. butyricum 10^8 CFU (n = 3); CB2 = C. butyricum 10^9 CFU (n = 3); CB3 = C. butyricum 10^{10} CFU (n = 3). Phyla with relative abundance in the bottom 1% were grouped under "Others"

Suppementary Table S2. Effects of C. butyricum on the rumen microbiota composition (genus level) in Hanwoo calves

Items (%)	CON	CB1	CB2	CB3	SEM	p-value
Acetitomaculum	2.05	0.42	0.65	0.82	0.31	0.281
Clostridia_UCG-014	5.16	6.82	6.78	4.65	0.71	0.675
Christensenellaceae_R-7_group	1.93	2.08	0.42	2.96	0.65	0.641
Defluviitaleaceae_UCG-011	1.29	1.92	1.78	1.49	0.43	0.968
F082	2.87	0.92	0.22	0.39	0.68	0.560
Lachnospiraceae_NK3A20_group	2.62	1.79	2.35	2.55	0.38	0.898
Muribaculaceae	2.95	0.50	4.63	7.52	1.00	0.056
NK4A214_group	1.79	2.12	0.58	5.46	1.09	0.489
p-251-o5	6.60	0.11	-	-	0.57	-
Prevotella	24.13	44.20	45.93	36.61	4.13	0.238
Prevotellaceae_UCG-001	1.87	1.57	2.10	1.74	0.35	0.971
Prevotellaceae_NK3B31_group	1.64	0.54	-	0.41	0.74	0.890
Rikenellaceae_RC9_gut_group	5.35	1.18	0.33	1.57	1.21	0.528
Ruminococcus	7.08	4.36	6.71	4.82	2.18	0.850
Saccharofermentans	1.16	0.65	0.34	0.67	0.25	0.748
Succiniclasticum	1.34	1.19	1.27	0.34	0.27	0.591
Syntrophococcus	3.75	3.53	0.78	2.22	0.76	0.548
Treponema	1.40	1.36	0.47	1.56	0.39	0.789
Others	30.00	25.86	24.65	24.48	2.18	0.838

CON = control group (no supplementation, n = 3); CB1 = C. butyricum 10^8 CFU (n = 3); CB2 = C. butyricum 10^9 CFU (n = 3); CB3 = C. butyricum 10^{10} CFU (n = 3). Genera with relative abundance in the bottom 20% were grouped under “Others”.

Suppementary Table S3. Effects of C. butyricum on the fecal microbiota composition (phylum level) in Hanwoo calves

Items (%)	CON	CB1	CB2	CB3	SEM	p-value
Actinobacteria	7.24	7.75	11.78	5.58	2.16	0.810
Bacteroidetes	10.61	12.09	11.41	31.95	4.03	0.177
Cyanobacteria	0.09	3.53	0.82	4.75	0.95	0.273
Firmicutes	63.76	68.45	70.35	52.26	0.453	0.535
Proteobacteria	18.24	8.08	0.48	5.33	0.391	0.470
Verrucomicrobiota	0.00	0.01	5.15	0.12	1.06	0.235
Others	0.06	0.09	0.01	0.01	0.03	0.560

CON = control group (no supplementation, n = 3); CB1 = C. butyricum 10^8 CFU (n = 3); CB2 = C. butyricum 10^9 CFU (n = 3); CB3 = C. butyricum 10^{10} CFU (n = 3). Phyla with relative abundance in the bottom 1% were grouped under "Others"

Suppementary Table S4. Effects of C. butyricum on the rumen microbiota composition (genus level) in Hanwoo calves

Items (%)	CON	CB1	CB2	CB3	SEM	p-value
Alloprevotella	4.62	0.40	0.34	7.39	1.90	0.523
Bacteroides	1.52	7.36	7.63	3.27	1.64	0.503
Blautia	2.16	1.89	2.72	1.00	0.69	0.874
Clostridia_UCG_014	6.79	4.04	2.25	8.54	1.57	0.538
Collinsella	4.45	3.71	5.61	2.67	1.27	0.894
Escherichia_Shigella	13.78	7.69	0.31	5.21	3.07	0.518
Faecalibacterium	8.69	8.09	8.31	7.17	2.33	0.997
Gastranaerophilales	0.09	3.67	0.74	5.61	1.15	0.305
Lactobacillus	11.12	7.20	9.49	7.82	2.26	0.943
Olsenella	0.49	0.44	3.50	1.67	0.77	0.497
Roseburia	0.03	0.49	1.25	0.19	0.31	0.558
Ruminococcus_torques_group	5.48	0.42	2.97	0.92	0.96	0.232
Ruminococcus	0.31	0.43	6.44	3.55	1.22	0.241
Tyzzerella	0.07	0.13	0.62	0.07	0.08	0.022
Others	40.42	54.04	47.80	44.89	2.97	0.465

CON = control group (no supplementation, n = 3); CB1 = C. butyricum 10^8 CFU (n = 3); CB2 = C. butyricum 10^9 CFU (n = 3); CB3 = C. butyricum 10^{10} CFU (n = 3). Genera with relative abundance in the bottom 20% were grouped under "Others".

Suppementary Figure S1. Principal coordinate analysis (PCoA) of ruminal microbiota in Hanwoo calves supplemented with *C. butyricum*

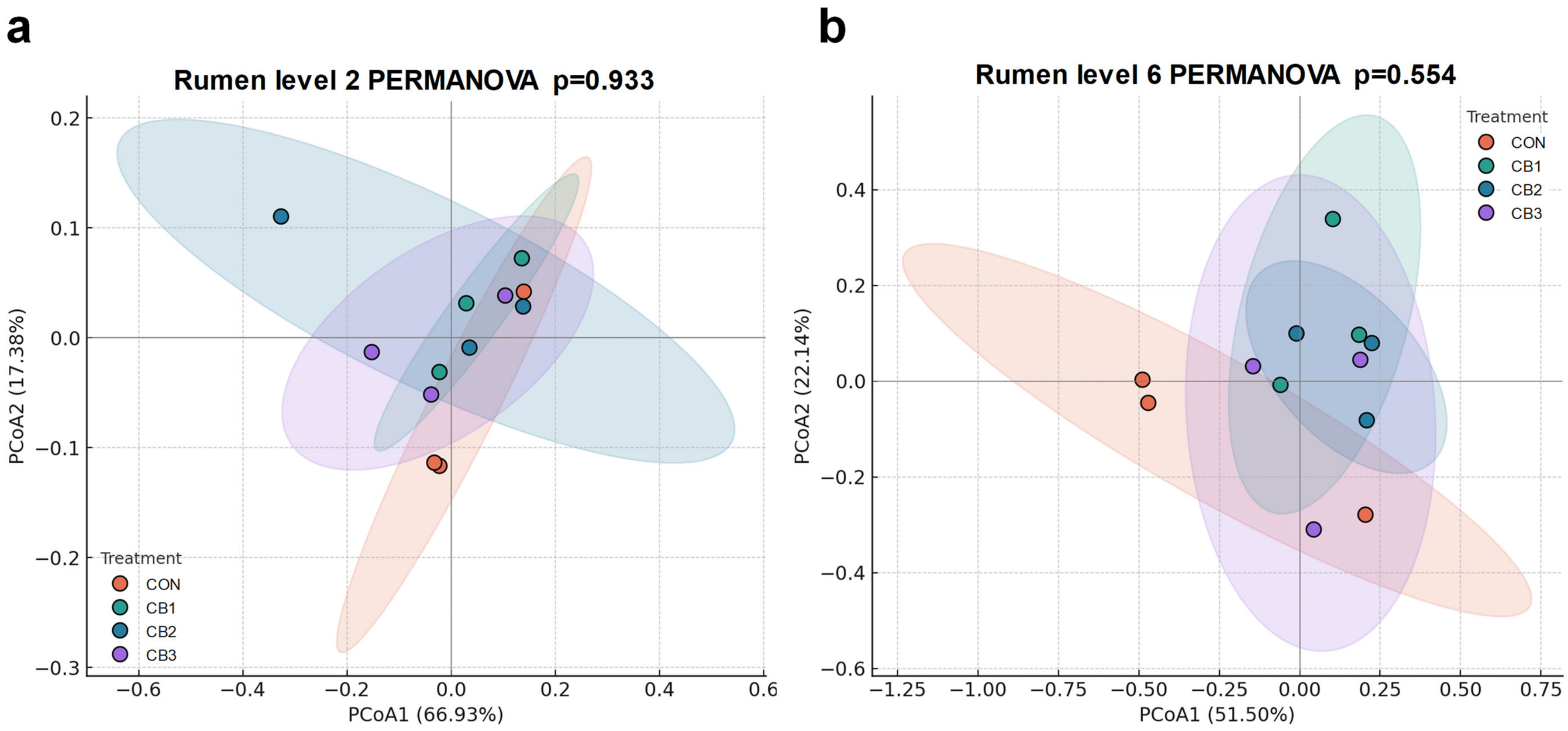

Principal coordinate analysis (PCoA) plots based on distances of ruminal microbial communities at (A) level 2 and (B) level 6 taxonomic resolution. Each point represents one calf, colored by treatment group: CON = control group (no supplementation, n = 3); CB1 = C. butyricum 10^8 CFU (n = 3); CB2 = C. butyricum 10^9 CFU (n = 3); CB3 = C. butyricum 10^{10} CFU (n = 3). Ellipses represent 95% confidence intervals for each group. PERMANOVA analysis indicated no significant differences in microbial community composition among groups (level 2, p = 0.933; level 6, p = 0.554).

Suppementary Figure S2. Principal coordinate analysis (PCoA) of fecal microbiota in Hanwoo calves supplemented with *C. butyricum*

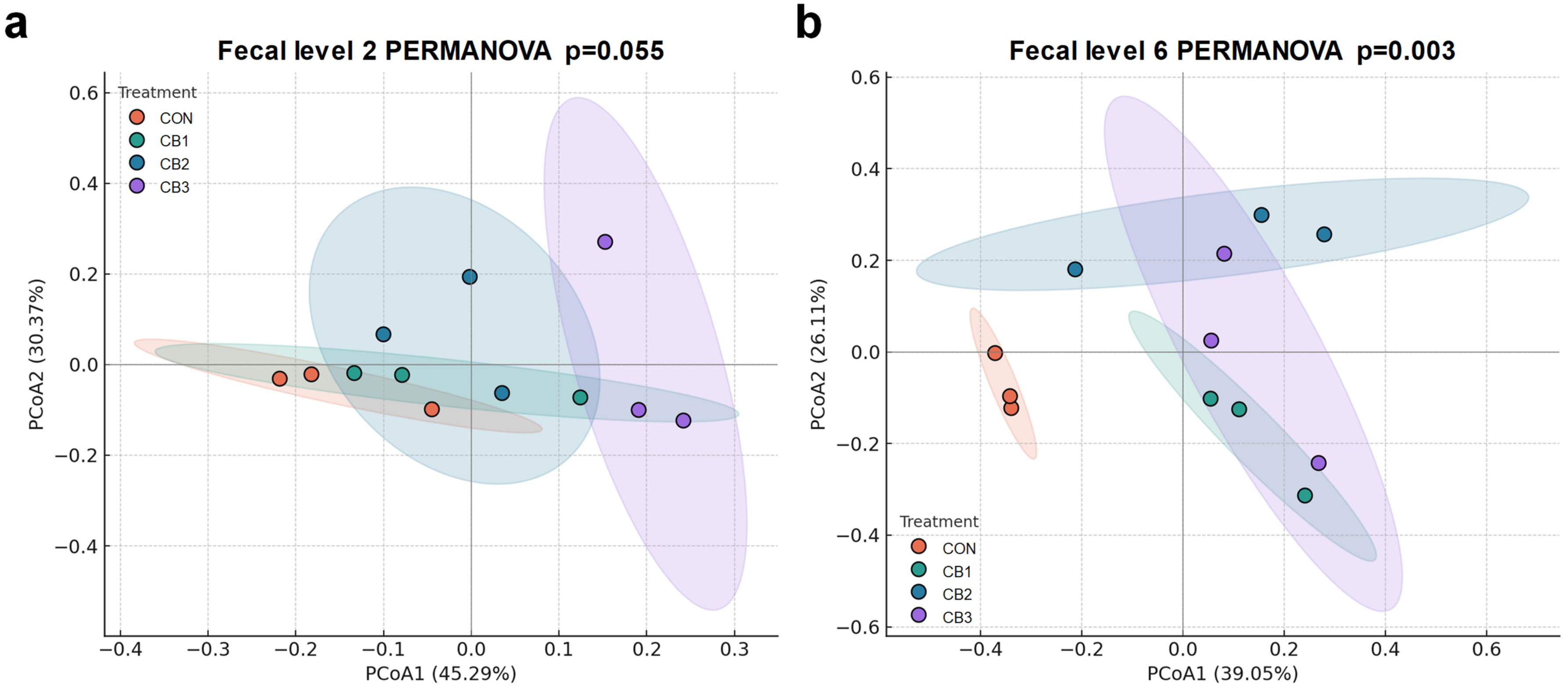

Principal coordinate analysis (PCoA) plots based on distances of fecal microbial communities at (A) level 2 and (B) level 6 taxonomic resolution. Each point represents one calf, colored by treatment group: CON = control group (no supplementation, n = 3); CB1 = C. butyricum 10^8 CFU (n = 3); CB2 = C. butyricum 10^9 CFU (n = 3); CB3 = C. butyricum 10^{10} CFU (n = 3). Ellipses represent 95% confidence intervals for each group. PERMANOVA analysis indicated no significant differences in microbial community composition among groups (level 2, $p = 0.055$; level 6, $p = 0.003$).
